# Comparison of Corvis ST Parameters between Primary Open-Angle Glaucoma and Primary Angle-Closure Glaucoma

**DOI:** 10.3390/jcm12155108

**Published:** 2023-08-03

**Authors:** Yuta Nakaniida, Kana Tokumo, Hiromitsu Onoe, Naoki Okada, Shunsuke Nakakura, Ryo Asaoka, Yoshiaki Kiuchi

**Affiliations:** 1 Department of Ophthalmology and Visual Science, Hiroshima University, 1-2-3 Kasumi, Minami-ku, Hiroshima 734-8551, Japan; 2 Department of Ophthalmology, Saneikai Tsukazaki Hospital, 68-1 Aboshi Waku, Himeji 671-1227, Japan; 3 Department of Ophthalmology, Seirei Hamamatsu General Hospital, 2-12-12 Sumiyoshi, Naka-ku, Hamamatsu 430-8558, Japan; 4 Seirei Christopher University, 3453 Mikatabara, Kita-ku, Hamamatsu 433-8558, Japan

**Keywords:** cornea, Corvis ST, glaucoma, PACG, POAG

## Abstract

Background: We compared corneal visualization Scheimpflug technology (CST) parameters between eyes with primary open-angle glaucoma (POAG) and primary angle-closure glaucoma (PACG). Methods: A retrospective analysis was performed on data from 89 eyes with POAG and 83 eyes with PACG that had CST examinations. CST parameters were compared between eyes with POAG and those with PACG using a linear mixed model (LMM). Results: No differences were observed in age, central corneal thickness, intraocular pressure, or use of antiglaucoma eye drops between the two groups. Patients with PACG had a significantly shorter axial length (AL), a higher proportion of females, CST parameters, longer applanation 2 (A2) time, deeper A2 deformation amplitude, shorter peak distance, longer whole eye movement, and longer whole eye movement time than patients with POAG. The highest concavity (HC) length and PD showed a significant positive correlation with AL. However, A1 length, A1 deformation amplitude, A2 time, A2 velocity, A2 length, A2 deformation amplitude, HC time, whole eye movement, and whole eye movement time were negatively correlated with AL. Conclusions: The biomechanical properties of the cornea differed between POAG and PACG. In some parts, AL differences between the POAG and PACG groups might contribute to the variation in CST parameters.

## 1. Introduction

Glaucoma is the second most common cause of blindness, affecting approximately 60 million people worldwide [1,2,3,4]. Glaucoma is defined as the loss of retinal ganglion cells that causes irreparable impairment of the visual field. Although increased intraocular pressure (IOP) is a well-established risk factor for glaucoma, recent research has uncovered other key factors influencing disease onset and progression. Several studies have suggested that glaucoma progression is related to central corneal thickness (CCT) magnitude. The thinner the CCT, the more likely the glaucoma is to progress [5,6]. Other biomechanical properties of the cornea affect glaucoma [7].

The corneal visualization Scheimpflug technology instrument (Corvis ST tonometry [CST]; Oculus, Wetzlar, Germany) uses an ultrahigh-speed Scheimpflug camera to quantify the biomechanical aspects of the cornea when a quick air puff is applied. As a result, the velocity of corneal deformation during the first and second applanations and the maximum depth of corneal deformation can be observed during the air puff application. Following the delivery of an air pulse, CST facilitates a thorough study of corneal deformities, and the characteristics of corneal behavior observed via CST are linked to glaucoma progression [7,8].

Previous studies on CST parameters have mostly focused on primary open-angle glaucoma (POAG) [7,8,9]. POAG is the most common type of glaucoma in the world [8]. POAG penetration rate is 3.5% [10]. There are other risk factors for POAG besides elevated IOP. Vulnerability at the optic disc, decreased blood flow in the tissue of the optic disc, and progression of myopia have also been reported as risk factors for POAG [11,12,13]. In POAG, eyes with short A1 time, short A2 time, long A2 length, and a deep highest concavity deformation amplitude are more likely to have progressive visual field damage due to glaucoma [7].

Primary angle-closure glaucoma (PACG) has anatomical features different from POAG [14,15]. This suggests that patients with PACG may have different CST parameter characteristics than patients with POAG. Elevated IOP secondary to angle closure is the basic disease mechanism of PACG [16,17]. Hyperopia is another risk factor for PACG [14,18,19]. Patients with PACG have a threefold higher risk of going blind compared to patients with POAG [20,21,22].

POAG and PACG differ significantly in clinical course. In POAG, the rate of visual field progression slows down as IOP is lowered [21,22]. However, there are individual differences in the rate of visual field progression. Furthermore, if the rate of visual field progression is fast in POAG, trabeculectomy is often performed to further reduce IOP, even if the IOP is in the normal range. On the other hand, the progression of PACG is based on elevated IOP [18]. This can be prevented if the angle closure process is resolved before irreversible trabecular damage occurs via laser peripheral iridotomy or cataract extraction. The optic disc often shows different features in POAG and PACG eyes. A higher prevalence of peripapillary atrophy has been reported in POAG [23]. On the other hand, in PACG, the optic papillae may become pallid [24].

There have been several studies comparing POAG and PACG [25,26,27], but there are no studies comparing the characteristics of CST parameters in POAG and PACG.

The purpose of this study is to compare CST parameters in patients with POAG and PACG.

## 2. Materials and Methods

This study was approved by the Ethics Committee for Clinical Research at Hiroshima University (E-826-4). Written consent was obtained from the patients for their information to be stored in the hospital database for use in this study. The study was conducted in accordance with the principles of the Declaration of Helsinki.

### 2.1. POAG and PACG Patients

This retrospective cross-sectional study investigated 172 eyes of 105 patients (89 eyes with POAG (54 patients) and 83 eyes with PACG (51 patients)). Data were retrospectively acquired at the Hiroshima University Hospital, Saneikai Tsukazaki Hospital, and Seirei Hamamatsu General Hospital between August 2019 and December 2021.

POAG was defined as (1) the presence of typical glaucomatous changes in the optic nerve head, including a rim notch with a rim width of ≤0.1 disc diameters or a vertical cup-to-disc ratio greater than 0.7 and/or a retinal nerve fiber layer defect with its width at the optic nerve head margin greater than a major retinal vessel, diverging in an arcuate or wedge shape, and (2) wide open angle with gonioscopy. POAG eyes with significant cataracts were excluded, except those with clinically insignificant senile cataracts on biomicroscopy.

PACG was defined as (1) the presence of angle closure, defined as at least 180° of the posterior pigmented trabecular meshwork not being visible on gonioscopy in the primary position of gaze without indentation, and (2) the existence of glaucomatous optic neuropathy, defined as neuroretinal rim loss with a vertical cup-to-disc ratio greater than 0.7 or between eye vertical cup-to-disc ratio asymmetry greater than 0.2, focal notching of the neuroretinal rim with a VF defect suggestive of glaucoma, or both.

Only patients aged ≥20 years were included in the study. Eyes with an IOP > 25 mmHg or a history of glaucoma attacks, laser treatment (iridotomy and selective laser trabeculoplasty; SLT), or ocular surgery, including glaucoma and cataracts, were excluded. If both eyes satisfied the inclusion criteria, the patients were included in the study.

Patients with PACG were referred from another hospital for laser or surgical treatment after receiving drug therapy only. The tests were performed before treatment.

### 2.2. Corvis ST Tonometer Measurement

The principles of CST are described in detail elsewhere [28]. Briefly, the camera recorded a sequence of images that captured corneal deformation following a rapid air puff. The device was capable of capturing 4330 images per second, which were analyzed to quantify CCT, applanation time, applanation length, corneal velocity, and deformation amplitude. Each measurement was further classified as follows: (1) A1/A2 time, (2) A1/2 length, (3) A1/2 velocity, and (4) A1/2 deformation amplitude. The applanation first (A1) time was the length of time from the initiation of the air puff to the first (when the cornea is moving inwards). The A1 length was the length of the flattened cornea at the first applanation. The A1 velocity was the velocity of the movement of the cornea during the first applanation. The A1 deformation amplitude was the deformation amplitude of the movement of the corneal apex of the flattened cornea at the first applanation. The applanation first (A2) time was the length of time from the initiation of the air puff to the second (when the cornea is moving outwards). The A2 length was the length of the flattened cornea at the second applanation. The A2 velocity was the velocity of the movement of the cornea during the second applanation. The A2 deformation amplitude was the deformation amplitude of the movement of the corneal apex of the flattened cornea at the second applanation. The highest concavity (HC) time was the length of time required to reach the highest concavity from the pre-deformation of the cornea. The HC length was the length of the flattened cornea at the HC from the pre-deformation of the cornea. The HC deformation amplitude was the magnitude of movement of the corneal apex from before deformation to its HC. The peak distance was the distance between two peaks surrounding the cornea at the HC. The radius was the central curvature radius at the point of the HC. The whole eye movement was the amplitude of the maximum whole eye movement. The whole eye movement time was the time of maximum whole eye movement.

CST (software version 1.6r2031) was performed three times on the same day with at least a one-minute interval. The average CST parameters (A1 time, A1 velocity, A1 length, A1 deformation amplitude, A2 time, A2 velocity, A2 length, A2 deformation amplitude, HC time, HC length, HC deformation amplitude, peak distance, radius, whole eye movement, and whole eye movement time) were calculated from three repeated tests. All CST measurements were considered reliable according to the “OK” quality index displayed on the device monitor.

### 2.3. Other Measurements

The CCT was measured using CST three times and the average of three times was used for analysis. The axial length (AL) was measured using an IOL Master 700 (Carl Zeiss Meditec, Dublin, CA, USA), and the average of three times was used for analysis. The IOP was measured using a Goldman applanation tonometer, and the average of three times was used for analysis.

### 2.4. Statistical Analysis

Continuous variables were presented as mean ± standard deviation and range. Categorical variables are presented as numbers and percentages.

A linear mixed model (LMM) was employed to compare the two groups in this study, considering the presence of two eyes per patient. In this study, age, AL, IOP, CCT, and CST parameters (A1 time, A1 velocity, A1 length, A1 deformation amplitude, A2 time, A2 velocity, A2 length, A2 deformation amplitude, HC time, HC length, HC deformation amplitude, peak distance, radius, whole eye movement, and whole eye movement time) were compared between POAG and PACG using a linear mixed model (LMM), considering the presence of two eyes per patient. LMM was equivalent to ordinary linear regression in that it described the relationship between predictor variables and a single outcome variable. However, standard linear regression analysis assumed that all observations were independent of each other. The measurements were nested within the patients and test points in this study; therefore, they were interdependent. Ignoring this grouping of measurements resulted in an underestimation of the standard errors of the regression coefficients. The LMM adjusted for the hierarchical structure of the data, modeling in a way in which measurements were grouped within patients to reduce the possible bias derived from the nested structure of the data [29,30].

The chi-square test was used to compare the proportions of males and females. The chi-square test is a test method for categorical variables. The chi-square tests include the chi-square test for goodness-of-fit, the chi-square test for independence, and the chi-square test for homogeneity. In this study, the chi-square test for homogeneity was used to compare the proportion of females of POAG and PACG.

Furthermore, Spearman’s rank correlation coefficient was used to correlate AL and CST parameters (A1 time, A1 velocity, A1 length, A1 deformation amplitude, A2 time, A2 velocity, A2 length, A2 deformation amplitude, HC time, HC length, HC deformation amplitude, peak distance, radius, whole eye movement, and whole eye movement time).

All statistical analyses were performed using the statistical programing language R (version 4.3.0; The Foundation for Statistical Computing, Vienna, Austria).

## 3. Results

The demographic information of POAG and PACG are shown in Table 1. The eyes with PACG had a significantly shorter AL than eyes with POAG (*p* < 0.001) and had a significantly higher proportion of females than the POAG group (*p* = 0.008). There were no significant differences in age, IOP, and CCT values between eyes with POAG and PACG (*p* = 0.85, 0.32, and 0.12, respectively).

The comparison of CST parameters between POAG and PACG are shown in Table 2. The A2 time in eyes with PACG was significantly longer than in eyes with POAG (*p* = 0.012). The A2 deformation amplitude in eyes with PACG was significantly greater than in eyes with POAG (*p* < 0.001). The peak distance in eyes with PACG was significantly shorter than eyes with POAG (*p* = 0.03,). The whole eye movement in eyes with PACG was significantly longer than in eyes with POAG (*p* < 0.001). The whole eye movement time in eyes with PACG was significantly longer than in eyes with POAG (*p* = 0.004). There were no significant differences in A1 time, A1 velocity, A1 length, A1 deformation amplitude, A2 time, A2 length, HC time, HC length, HC deformation amplitude, and Radius values between eyes with POAG and PACG (*p* = 0.36, 0.47, 0.053, 0.17, 0.068, 0.79, 0.24, 0.14, 0.58, and 0.76, respectively).

The correlation with axial length is shown in Table 3. The HC length and peak distance had a significant positive correlation with AL (r = 0.35, *p* < 0.001, and r = 0.41, *p* < 0.001, respectively). On the other hand, A1 length, A1 deformation amplitude, A2 time, A2 velocity, A2 length, A2 deformation amplitude, HC time, whole eye movement, and whole eye movement time showed a significant negative correlation with AL (r = −0.18, *p* = 0.004; r = −0.25, *p* < 0.001; r = −0.16, *p* = 0.01; r = −0.27, *p* < 0.001; r = −0.18, *p* = 0.005; r = −0.61, *p* < 0.001; r = −0.30, *p* < 0.001; r = −0.60, *p* < 0.001; and r = −0.31, *p* < 0.001, respectively). A1 time, A1 velocity, HC deformation amplitude and radius were not significantly correlated with AL (r = 0.04, *p* = 0.53; r = −0.02, *p* = 0.72; r = −0.02, *p* = 0.73; and r = 0.10, *p* = 0.11, respectively).

## 4. Discussion

In this study, we compared CST parameters between eyes with POAG and those with PACG. The PACG group had a shorter AL and a higher proportion of females compared to the POAG group. The backgrounds of the patients in this study were consistent with those of many previous reports [14,15,16,17]. A longer AL is more likely to deform the cornea. Higher IOP results in scant corneal deformation [1], and age resists both corneal deformation and IOP [31]. In this study, no significant difference was observed between age and IOP. Many non-contact tonometers convert A1 time to IOP. The fact that A1 time and IOP values did not differ between POAG and PACG is consistent with theory. In PACG, IOP are highly variable and do not always show a punctuated IOP at the time of examination. In PACG, the CCT has been reported to be significantly thicker [32], thinner [33], or not significantly different [34] from POAG. Moreover, no significant differences were observed in the CCT.

In this study, we observed a significant difference in AL, but not in IOP. This suggests that IOP does not depend on differences in AL. Previous study has reported that higher IOP is associated with lower peripapillary vessel density in glaucoma patients with a longer AL [35].

Significant differences were observed between the POAG and PACG groups regarding the CST parameters for corneal movement. Compared to POAG, PACG had a longer A2 time, deeper A2 deformation amplitude, shorter peak distance, longer whole eye movement, and longer whole eye movement time. These parameters were affected by the AL. A2 time, A2 deformation amplitude, whole eye movement, and whole eye movement time decreased with increasing AL. On the other hand, the peak distance increased with increasing AL. The difference in CST parameters between POAG and PACG was most likely due to the difference in AL. No significant difference was observed in the CST parameters between the POAG and PACG groups when the corneal depression phase started with an air puff. The A1 time is when the corneal surface first flattens after the cornea begins to depress. Non-contact tonometer converts A1 time into IOP. The fact that the A1 time, which is important for IOP measurement, was not affected by IOP and AL means that in actual clinical practice, differences in AL and disease type do not need to be considered when interpreting IOP measurement results.

Corneal biomechanics has been reported to influence glaucoma progression. Previous studies [7] have shown that the cornea begins to deform faster and returns from deformation with a faster progression of visual field loss. Glaucomatous visual field defects in POAG progress more rapidly in patients with corneal deformities and return quickly after being pushed into the cornea [7]. The A2 time was the length of time from the initiation of the air puff to the second (when the cornea is moving outwards). The A2 deformation amplitude was the deformation amplitude of the movement of the corneal apex of the flattened cornea at the second applanation. It has been reported that higher IOP results in a shorter A2 time and shallower A2 deformation amplitude. A longer A2 time and deeper A2 deformation amplitude in PACG indicate that the return of corneal deformation is faster in POAG than in PACG. Glaucomatous visual field defects are more likely to progress if the eye returns more quickly after being pushed into the cornea. POAG is considered more prone to progression when considering A2 time and A2 deformation amplitude. However, glaucoma is a multifactorial disease, and the pathogenetic mechanisms of PACG and POAG are completely different. The tendency for visual field defects to progress more rapidly in patients with corneal deformities may be a feature of POAG and not of PACG. Therefore, it is not possible to conclude that POAG is more prone to progressive glaucomatous optic neuropathy than PACG. It has been reported that higher IOP results in a longer A1 time, slower A1 velocity, shorter A2 time, faster A1 velocity, and shallower HC deformation amplitude [8]. However, in this study, the A2 time was longer, and the A2 deformation amplitude was deeper in PACG, with no significant difference in IOP. This suggests that PACG has biomechanical characteristics that make the cornea more concave and less likely to return to the outwards. The significant negative correlation with AL may be due to differences in ocular structure between POAG and PACG.

The peak distance is the distance between the two highest points of the cornea at the maximum depression. In a hard cornea, the peak distance is smaller than that in a soft, thin cornea at the same IOP. In this study, there was no significant difference in IOP and CCT, and the peak distance was shorter in PACG, suggesting that the cornea of PACG is stiffer than that of POAG. The peak distance is reported to be shorter in males [8]. In this study, the proportion of females was higher in PACG, but there was no significant difference in peak distance. The reason for this difference may be that in the previous study, the subjects were normal eyes, and the previous study was to determine the relationship between CST parameters (A1 time, A1 velocity, A1 length, A2 time, A2 velocity, A2 length, HC time, HC deformation amplitude, peak distance) and basic parameters (age, sex, AL, IOP, CCT), whereas this study compared CST parameters (A1 time, A1 velocity, A1 length, A1 deformation amplitude, A2 time, A2 velocity, A2 length, A2 deformation amplitude, HC time, HC length, HC deformation amplitude, peak distance, radius, whole eye movement, and whole eye movement time) between POAG and PACG. Therefore, the peak distance was no longer in PACG with a significantly higher proportion of females.

The whole eye movement and whole eye movement time represents the backward displacement and time of the eyeball calculated from the corneal periphery (4 mm away from the corneal apex in each horizontal direction). Tonometry has shown that the backward displacement and time of the eye is greater in elderly females than in males or young adults. The fact that whole eye movement and whole eye movement time were greater in the PACG with a higher proportion of females in this study is consistent with previous reports [34]. The eyes that move backward under air pressure for shorter distances and time are closely related to glaucoma [36]. Longer whole eye movement and longer whole eye movement time in PACG, indicating that POAG is more prone to glaucoma progression than PACG. However, it is possible that this tendency may also be a feature of POAG. The characteristics of the CST parameters of PACG, which are more likely to progress, are unknown.

Collectively, the corneal parameters of the CST suggest that POAG is more prone to glaucoma progression than PACG. However, the reported characteristics of CST parameters that are more prone to progression are from studies of POAG, and the characteristics of POAG and PACG that are more prone to progression of visual field damage are likely to be different. Because, the pathogenetic mechanisms of PACG and POAG are completely different. In addition, PACG are usually much higher than those in POAG [14,15,16,17]. Furthermore, PACG has a shorter AL than POAG, and the ocular structures are very different. In addition, patients with POAG and PACG also have different demographic information, such as a higher proportion of females.

From the perspective of CST parameters, POAG has the characteristics of POAG, which is more prone to progression in previous reports, compared to PACG. In previous reports, PACG has been reported to have a worse prognosis than POAG, with a three-fold higher risk of blindness. This may have been due to the glaucoma attacks and higher IOP. This suggests that the CST parameters of POAG and PACG are different, and the characteristics of CST parameters for POAG and PACG with progressive visual field impairment may also be different.

Yousefi et al. [25] excluded eyes with a history of glaucoma attacks, laser-treated eyes, and eyes with a history of cataract surgery. They examined patients with relatively stable IOP who received eye drop therapy, which may explain why no difference was observed in the progression rate between POAG and PACG. Furthermore, both POAG and PACG eyes showed a similar tendency in characteristics, with visual field damage being more pronounced in superior hemifield than inferior hemifield. Although the tendency of visual field damage is similar in POAG and PACG, the characteristics of CST parameters are different. This suggests that treatment and prevention approaches for POAG and PACG may differ. Characterization of CST parameters of POAG and PACG, which are prone to progression, respectively, can contribute to understanding the pathogenesis of POAG and PACG, preventing their development, and slowing their progression.

No difference was observed in the shape of the optic nerve head between POAG and PACG when the degree of visual field progression was matched with the optical coherence tomography (OCT) findings. The optic nerve shape was evaluated using Heidelberg retina tomography, OCT, and nerve fiber layer (NFL) examination [37]. No difference was observed in the speed of progression of the visual field between the POAG and PACG groups. Although the superior half of the visual field progressed faster than the inferior half in POAG, no difference was observed between the superior and inferior halves in PACG [25].

This study had several limitations. First, we compared the CST parameters (A1 time, A1 velocity, A1 length, A1 deformation amplitude, A2 time, A2 velocity, A2 length, A2 deformation amplitude, HC time, HC length, HC deformation amplitude, peak distance, radius, whole eye movement, and whole eye movement time) between eyes with POAG and PACG. However, the relationship between functional changes in visual field testing and morphological changes in OCT and CST parameters (A1 time, A1 velocity, A1 length, A1 deformation amplitude, A2 time, A2 velocity, A2 length, A2 deformation amplitude, HC time, HC length, HC deformation amplitude, peak distance, radius, whole eye movement, and whole eye movement time) could not be determined. Second, most POAG and PACG patients take antiglaucoma eye drops to control IOP and do not consider the effect of antiglaucoma eye drops on the biomechanical properties of the cornea [38,39,40,41]. Finally, normal eyes were not included in this study. Thus, it is unclear to what extent the CST parameters (A1 time, A1 velocity, A1 length, A1 deformation amplitude, A2 time, A2 velocity, A2 length, A2 deformation amplitude, HC time, HC length, HC deformation amplitude, peak distance, radius, whole eye movement, and whole eye movement time) of POAG and PACG differ from those of normal eyes.

## 5. Conclusions

In conclusion, the biomechanical properties of the cornea differed between POAG and PACG. AL differences between the POAG and PACG groups do not affect IOP, but they may partially contribute to the variation in CST parameters. Differences in CST parameters due to AL differences between POAG and PACG groups are useful for underlying the pathogenesis, prevention, and treatment of POAG and PACG.

## Figures and Tables

**Table 1:** Demographic information of POAG and PACG.

Parameters	POAG	PACG	*p*-Value
Patients	54	51	-
Eyes	89	83	-
Age (years)	72.02 (5.91)	72.52 (9.95)	0.85
Sex (% female)	26/28 (51.8%)	13 /38 (74.5%)	0.008
AL (mm)	24.78 (1.41)	23.05 (1.08)	<0.001
IOP (mmHg)	15.45 (3.36)	16.1 (3.74)	0.32
CCT (μm)	530.39 (29.92)	538.94 (32.51)	0.12

*p*-values were calculated by applying linear mixed models to compare POAG and PACG groups. Data represent mean (standard deviation). Abbreviations: POAG, primary open-angle glaucoma; PACG, primary angle-closure glaucoma; AL, axial length; IOP, intraocular pressure; CCT, central corneal thickness.

**Table 2:** Comparison of CST parameters between POAG and PACG.

Parameters	POAG	PACG	*p*-Value
A1 time	7.41 (0.35)	7.36 (0.34)	0.36
A1 velocity	0.15 (0.02)	0.15 (0.02)	0.47
A1 length	2.3 (0.17)	2.38 (0.14)	0.053
A1 deformation amplitude	0.13 (0.01)	0.14 (0.01)	0.17
A2 time	21.51 (0.55)	21.76 (0.49)	0.012
A2 velocity	−0.27 (0.04)	−0.26 (0.03)	0.068
A2 length	2.97 (0.61)	2.92 (0.50)	0.79
A2 deformation amplitude	0.43 (0.09)	0.51 (0.09)	<0.001
HC time	16.98 (0.60)	17.08 (0.46)	0.24
HC length	6.39 (0.63)	6.24 (0.56)	0.14
HC deformation amplitude	1.10 (0.10)	1.11 (0.10)	0.58
Peak distance	4.98 (0.33)	4.84 (0.33)	0.03
Radius	6.83 (0.67)	6.79 (0.84)	0.76
Whole eye movement	0.33 (0.09)	0.40 (0.09)	<0.001
Whole eye movement time	21.74 (0.87)	22.17 (0.84)	0.004

*p*-values were calculated by applying linear mixed models to compare POAG and PACG groups. Data represent mean (standard deviation). Abbreviations: POAG, primary open-angle glaucoma; PACG, primary angle-closure glaucoma; A1, applanation first; A2, applanation second; HC, highest concavity; CST, corneal visualization Scheimpflug technology.

**Table 3:** Correlation with axial length.

Parameters	r	*p*-Value
A1 time	0.04	0.53
A1 velocity	−0.02	0.72
A1 length	−0.18	0.004
A1 deformation amplitude	−0.25	<0.001
A2 time	−0.16	0.01
A2 velocity	−0.27	<0.001
A2 length	−0.18	0.005
A2 deformation amplitude	−0.61	<0.001
HC time	−0.30	<0.001
HC length	0.35	<0.001
HC deformation amplitude	−0.02	0.73
Peak distance	0.41	<0.001
Radius	0.10	0.11
Whole eye movement	−0.60	<0.001
Whole eye movement time	−0.31	<0.001

*p*-values were calculated by applying Spearman’s rank correlation coefficient. Abbreviations: A1, applanation first; A2, applanation second; HC, highest concavity.

## Data Availability

The data analyzed in this study are available from the corresponding author on reasonable request.
